# Atypical Placental Site Nodules within the Diverticulum of the Uterine Incision, a Rare Gestational Trophoblastic Disease Misdiagnosed as Intrauterine Residue: A Case Report

**DOI:** 10.1007/s43032-023-01361-2

**Published:** 2023-10-02

**Authors:** Xin Li, Yanli Li, Xiuting Shi, Shiyu Cheng, Tingzhu Meng, Han Gao, Jie Shi

**Affiliations:** 1 Medical College, Wuhan University of Science and Technology, Wuhan, 430065 People’s Republic of China; 2 Department of Gynecology, Maternal and Child Health Hospital of Hubei Province, Tongji Medical College, Huazhong University of Science and Technology, Wuhan, 430070 People’s Republic of China

**Keywords:** Atypical placental site nodule (APSN), Uterine incision, Diverticulum, Intrauterine residue, Misdiagnosis

## Abstract

Atypical placental site nodule (APSN) is a rare benign gestational trophoblastic disease (GTD). It is a tumor-like transformation that has a certain probability of developing into a placental site trophoblastic tumor (PSTT) or epithelioid trophoblastic tumor (ETT). Because of its atypical clinical presentation, it is difficult to diagnose and susceptible to misdiagnosis highly, thus delaying the patient’s condition. We report a scarce case of atypical nodules at the placental site of the uterine incision diverticulum in a 35-year-old female, who was irregular vaginal bleeding after a cesarean Sect. 2 years. She was diagnosed by several local hospitals with intrauterine residue and was given a variety of Traditional Chinese Medicine (TCM) orally, but the symptoms of irregular vaginal bleeding have not been alleviated. After being transferred to several hospitals, she went to Hubei Maternal and Child Health Hospital for treatment. Under the condition of excluding the second pregnancy, she underwent hysteroscopic resection of lesions and laparoscopic repair of uterine incision diverticulum. The pathological diagnosis after the operation suggested that the focus at the uterine incision was an atypical placental nodule that invaded the myometrium of the uterus. The operation completely removed the focus, and then the patient was followed up every 3 months in the first postoperative year, then every 6 months up to 3 years, and then annually thereafter up to 5 years, and then maybe every 2 years thereafter. The patient’s condition was quickly controlled, and the prognosis was good.

## Introduction

In 2020, the World Health Organization classified gestational trophoblastic disease (GTD) according to histology, which includes gestational trophoblastic neoplasia (GTN) and tumor-like lesions [1]. Placental site trophoblastic tumor (PSTT) and epithelioid trophoblastic tumor (ETT) are both GTNs composed of intermediate trophoblasts, which are clinically seldomness than choriocarcinoma and only account for 2 to 3% of overall GTN [2]. Placental site nodules (PSN) are tumor-like lesions with a histologic composition very similar to PSTT/ETT and are rarely seen clinically [2, 3]. Atypical placental site nodules (APSN) are even rarer, and related literature has seldom been reported.

After implantation, trophoblasts can differentiate into cytotrophoblasts (CT) and syncytiotrophoblasts (ST). Most studies suggest that the ST differentiates from the CT and that the intermediate trophoblasts (IT) are the transitional cells for this change. The IT can be divided into three subtypes, in which the implantation site IT can invade the decidua and myometrium, and this aberrant value added can lead to carcinoma. PSTT and PSN are both composed of IT. It has been reported that there is a 10%~15% probability of APSN coexisting with or progressing to PSTT/ETT [4].

Microscopically, these abnormal cell clusters are often composed of diffusely uniform mononuclear cells, multinucleated cells are rare, and nuclear fission is variable. The cells may be single or fused into sheets, strips, or islands. Immunohistochemically, the PSTT may show a relatively characteristic strong positivity for hPL and weak positivity for hCG, in addition to trophoblastic tumor markers [5]. PSTT proliferative indices are usually high, with Ki-67 labeling indices of approximately 10–30%. The majority of lesion cells in PSN express PLAP and p63, while hPL and MCAM (CD146) are often negative. The trophoblast cells of PSN have low proliferative activity, and the Ki-67 labeling index is less than 8% [5]. According to the current study, APSN cytologic structure is between PSN and ETT.

The clinical manifestations of APSN are atypical, which can lead to misdiagnosis easily and delay treatment. APSN mainly occurs in women of childbearing age [6]. Once the disease is delayed and continues to develop into PSTT/ETT, many women of childbearing age are likely to face a hysterectomy, which has a great impact on subsequent fertility [7]. Therefore, the disease needs to be paid attention to.

The diagnostic criteria for APSN have not yet been defined in the latest guidelines for the management of GTD [1]. APSN is seldom reported, and it is located in the endometrium of the uterine body and has not invaded the myometrium mostly in the related literature [8, 9]. In this study, we report a case of APSN located in the uterine diverticulum after cesarean section, which was misdiagnosed as intrauterine residue, and we found that this case of APSN invaded the myometrium of the uterus.

## Description of case

**Fig. 1:**
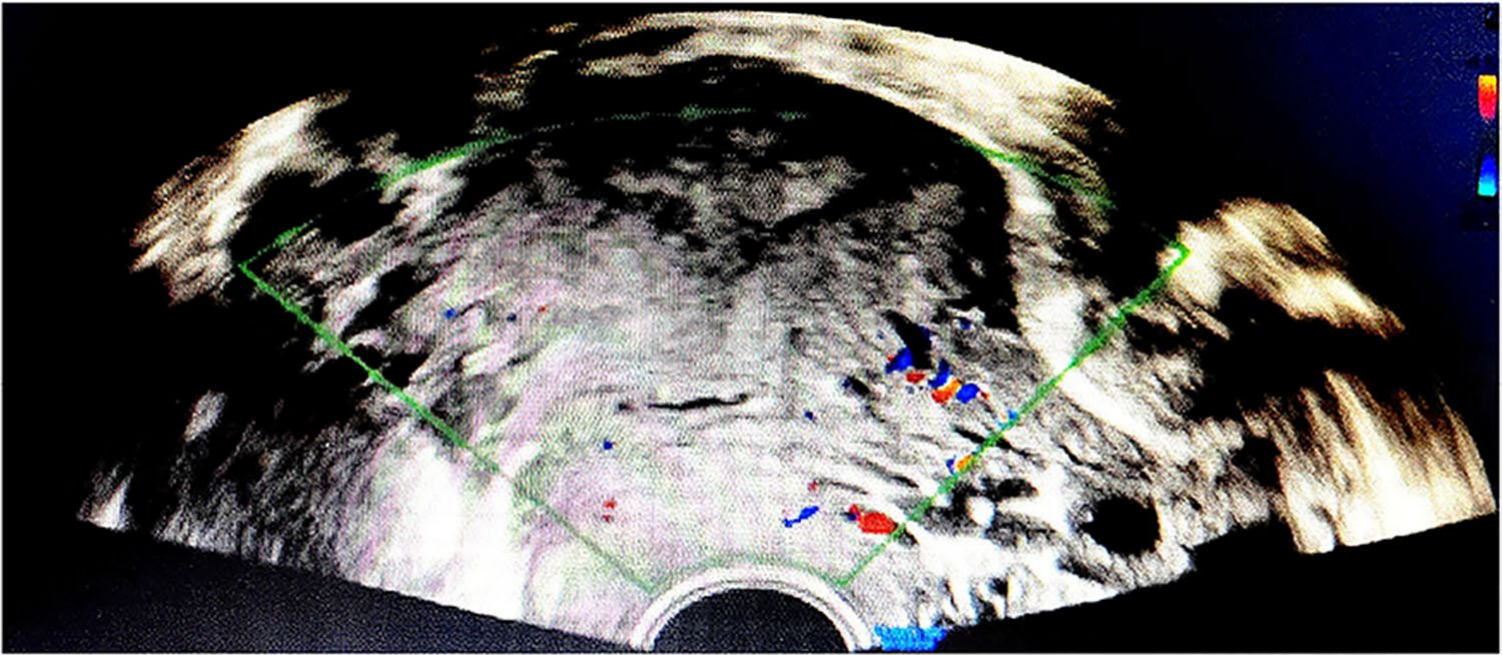
Doppler examination revealed a 1.4 × 2.0 × 0.7 cm hypoechoic area was observed at the incision, with uneven internal echo, communication with the uterine cavity, and visible blood flow signals around

A 35-year-old female presented with irregular vaginal bleeding for 1 year after a cesarean section. Reviewing the patient’s medical history, she had four previous pregnancies, which ended in induced abortion, induction of labor at 18 weeks of gestation due to “fetal demise,” biochemical pregnancy, and cesarean section in September 2020 at 38 weeks of gestation due to breech presentation of the fetus. No low-lying placenta was found in any of the four pregnancies. The patient had no history of abnormal uterine bleeding, and the previous history did not suggest endometrial lesions. She passed through several local hospitals for treatment, and all of them considered the intrauterine residue after delivery and rendered Chinese patent medicine oral symptomatic treatment. After many visits, the patient’s state of illness has not been significantly relieved. Then, the patient was treated at Hubei Maternal and Child Health Hospital. A review of this patient's laboratory tests showed a negative value of serum β-HCG. These laboratory tests and color Doppler ultrasound ruled out the possibility of pregnancy again. The color Doppler ultrasound showed a 1.4×2.0×0.7cm hypoechoic area at the incision, with uneven internal echo, communication with the uterine cavity, and visible blood flow signals around (Fig. 1).

**Fig. 2:**
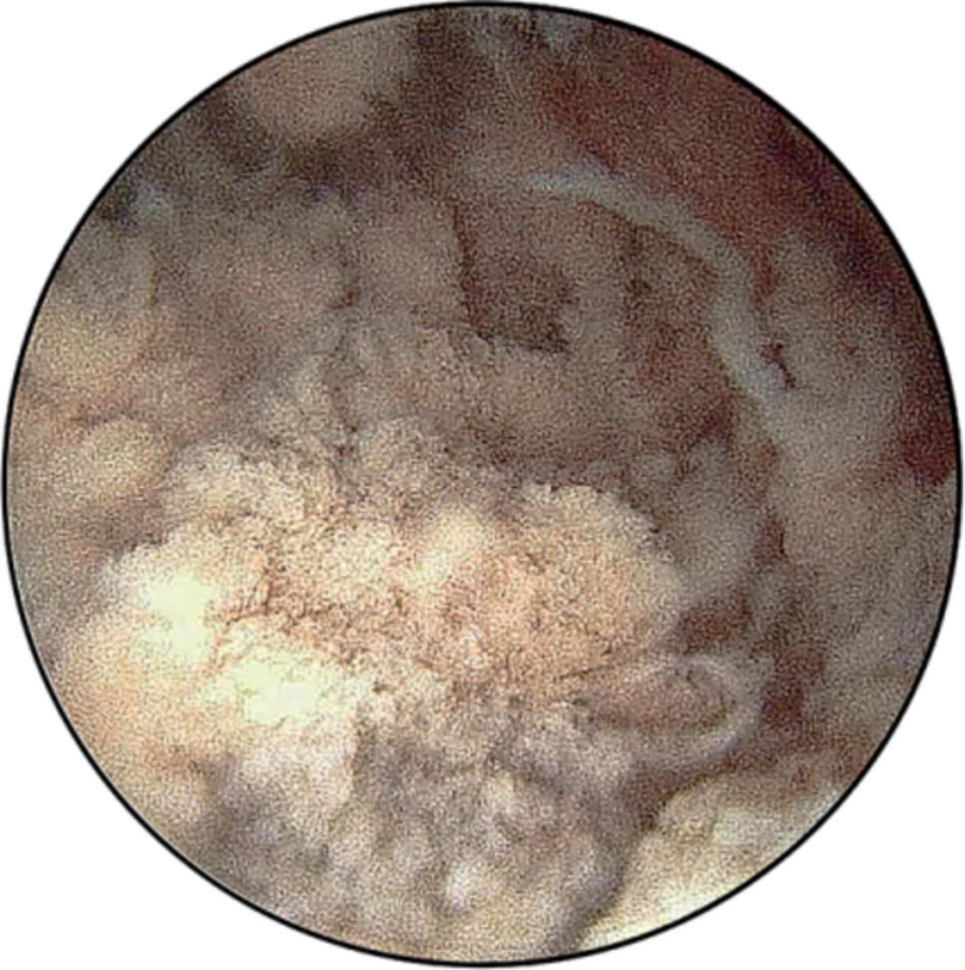
Intraoperative shows old villi-like tissue tight adhering to the uterine incision diverticulum

Combined with the patient’s medical history, laboratory examination, and imaging inspection, we decided to take hysteroscopy to investigate the specific situation in the uterine cavity. The intraoperative finds old villi-like tissue tight adhering to the uterine incision diverticulum (Fig. 2). We tried to scrape off these old villous tissues. Because the adhesion was dense excessively, and the possibility of implantation was not obviated, the operation was extremely difficult.

**Fig. 3:**
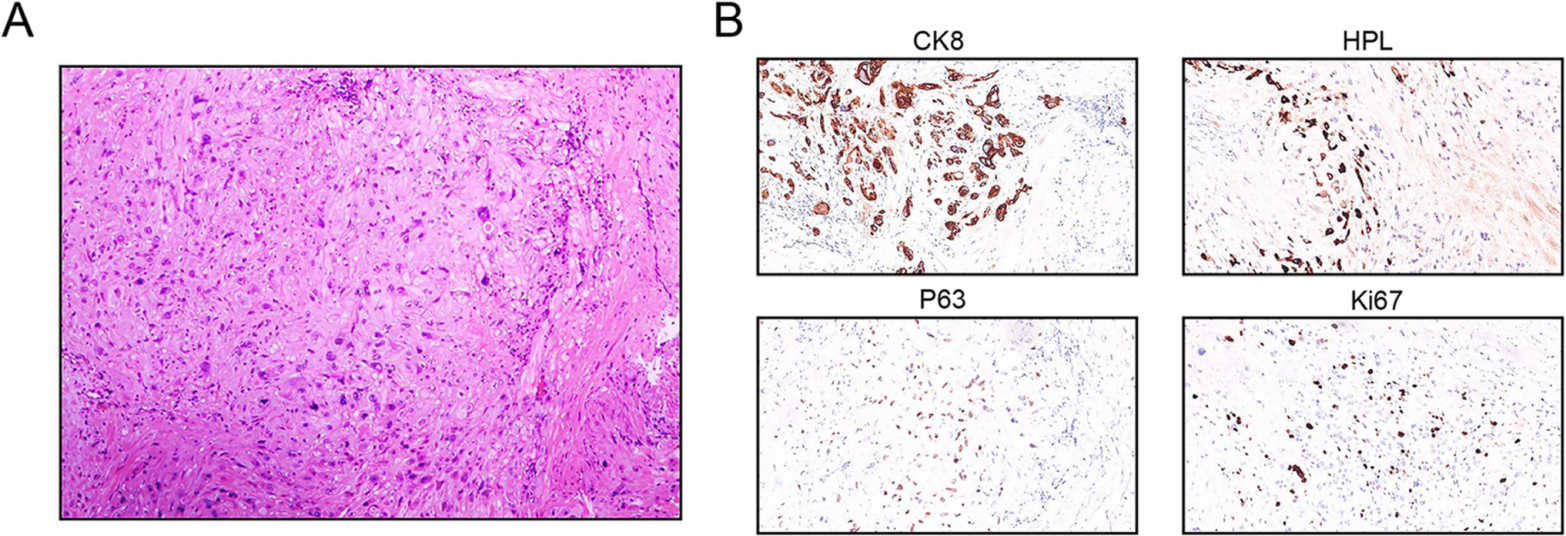
Atypical placental site nodule (APSN). Markedly typical smooth muscle cells, intermediate-type trophoblast cells, and eosinophilic stroma in a curettage specimen (**A**). Immunohistochemistry shows CK8( +), HPL ( +), P63(partial +), and Ki67(LI: hot spot area about 10%) (**B**)

Fully communicate the condition and risk, and the patient signs the informed consent form for the operation. Then she underwent a hysteroscopic resection of uterine lesions and laparoscopic repair of the uterine incision diverticulum. The operation was successful, and the intraoperative bleeding was 20ml. The pathological section of the uterine lesions shows that the lesion tissue is about 20mm*20mm*5mm, with smooth muscle cells, intermediate-type trophoblast cells, and eosinophilic stroma. Immunohistochemistry suggested: CK8(+), GATA-3(+), HPL (+), P63(partial +), CD146(partial +), β-HCG (-), Ki67(LI: hot spot area about 10%) (Fig. 3). According to the patient’s pathological findings and immunohistochemistry, APSN was diagnosed by two pathologists.

The patient was discharged after no vaginal bleeding from the third postoperative day and all health indicators were within normal limits on recheck. After 6 months of follow-up, the patient’s menstrual cycle returned to normal regularity with no abnormal vaginal bleeding, and the patient was advised to follow up for life to prevent the progression of the disease.

## Discussion

The concept of “placental site nodule or plaque (PSNP)” was first reported by Young RH, and Kurman RJ in 1990. This conception continued to be cited in subsequent clinical and pathological studies. The old view suggests PSN is a benign non-neoplastic lesion that does not require specific clinical treatment or even follow-up. However, as research continues to progress, the World Health Organization (WHO) 2020 (5th edition) classification criteria for the pathology of female genital tumors classify PSNP and super-normal placental site reflections as tumor-like lesions in GTD, because some studies have found that 10 to 15% of APSN will coexist with or progress to PSTT/ETT, there is a need to improve the understanding of APSN in clinical and pathological aspects [1].

This case reported a female was 35 years old and had a history of cesarean section and was admitted with long-term abnormal vaginal bleeding. There were no other specific clinical manifestations. Local hospitals keep misdiagnosing it as intrauterine residue. The intrauterine lesion was not detected until a hysteroscopy was performed. The present report suggests the necessity to hysteroscopy to avoid wrong subsequent treatments, prevent a delay in diagnosis, reduce misdiagnosis, and improve the survival rate of patients.

APSN, PSTT, ETT, and intrauterine residuals all occur in women of gestational age, and all present with irregular vaginal bleeding, making them clinically indistinguishable and often requiring pathologic differentiation [2].

PSN, ETT, and PSTT are all trophoblastic diseases, and all express cytokeratins, HLA-G, and GATA-3. Most diseased cells in PSN express PLAP and p63, whereas hPL and MCAM (CD146) are often negative [5]. Trophoblast proliferative activity is low in PSN, so the Ki-67 labeling index is often less than 8%. Cyclin-e positivity is seen in some APSNs and ETTs, whereas PSN usually lacks expression. Immunohistochemical findings in ETT often show diffuse expression of EMA, Cyclin-e, p63, and PLAP, while MCAM (CD146), hCG, and hPL are weakly and focally expressed [5]. P63 is strongly positive in ETT and is often used to differentiate it from other malignant trophoblastic tumors. The trophoblast proliferative activity of ETT is high, so the Ki-67 labeling index is often greater than 10%. The majority of cells in PSTT express hPL and MCAM (CD146), with occasional positive expression of PLAP and p63, which can distinguish PSTT from ETT [4]. HCG and inhibin expression is restricted to multinucleated cells. The proliferative index is usually higher compared to PSN, with a Ki-67 labeling index of about 10–30%.

Combining the above immunohistochemical results, p63, CD146, KI-67, and hPL can be used as the main basis for identifying the three diseases, with the Ki-67 labeling index being the most important. This patient had hPL (+), P63 (partial +), CD146 (partial +), β-HCG (-), and Ki67 (LI: hot spot area about 10%). In conclusion, this patient meets the diagnostic criteria of PSN, but the Ki67 value-added index is between PSN and PSTT/ETT. Combined with morphology, immunohistochemistry, and clinical history, it can be diagnosed as APSN with malignant tendency and more inclined to PSTT [10]. APSN can be considered a transition state between typical PSN and PSTT/ETT, and the diagnostic criteria are still unclear. Further case generalization studies are required. The diagnosis and treatment of APSN need to be specified and applied to the clinic [11].

The most difference between the patient in this study and the APSN patients reported in the past is that the pathological section of the uterine lesions shows smooth muscle cells. This indicates that the diseased tissue has invaded the muscular layer, which has not been reported in the relevant literature.

The patient needs to follow up, and once the lesion escalates, it can be detected, diagnosed, and treated as soon as possible. In this case, the follow-up time was short, and no signs of lesion escalation were found.

Based on this case, we consider combined hysteroscopy and laparoscopy management to be the best diagnostic and treatment modality for patients with APSN. The pathologic classification of placental nodules includes typical and atypical placental nodules. To further differentiate between these two types of nodules, surgery is required to make a definitive diagnosis based on the results of the pathologic tests. Hysteroscopy allows direct visualization of the lesions in the uterine cavity. Laparoscopic surgery allows complete excision of the lesion and immediate repair of uterine perforation if it occurs. In addition, laparoscopy can determine whether the lesion invades the myometrium or serosa or whether it invades other pelvic organs.

## Data Availability

Not applicable.
